# Controlled Preparation of Thermally Stable Fe-Poly(dimethylsiloxane) Composite by Magnetic Induction Heating

**DOI:** 10.3390/polym10050507

**Published:** 2018-05-07

**Authors:** Laila M. Al-Harbi, Mohamed S. A. Darwish, Manal M. Khowdiary, Ivan Stibor

**Affiliations:** 1 Chemistry Department, King Abdulaziz University, 80203 Jeddah, Saudi Arabia; lalhrbi@kau.edu.sa; 2 Egyptian Petroleum Research Institute, 1 Ahmed El-Zomor Street, El Zohour Region, Nasr City, Cairo 11727, Egypt; 3 Institute for Nanomaterials, Advanced Technologies and Innovation, Technical University of Liberec, Studentská 2, 461 17 Liberec, Czech Republic; ivan.stibor@tul.cz; 4 Chemistry Department, Lieth University Collage, Umm el Qura University, 2207 Mecca, Saudi Arabia; mmkhowdiary@uqu.edu.sa

**Keywords:** PDMS composites, induction heating, thermal stability

## Abstract

The most challenging task in the preparation of poly(dimethylsiloxane) composites is to control the curing time as well as to enhance their thermal and swelling behavior. Curing rate can be modified and controlled by a range of iron powder contents to achieve a desired working time, where iron is used as self-heating particles. Iron under alternative current magnetic field (ACMF) is able to generate thermal energy, providing a benefit in accelerating the curing of composites. Three types of iron-Poly(dimethylsiloxane) (Fe-PDMS) composites were prepared under ACMF with iron content 5, 10, and 15 wt %. The curing process was investigated by FTIR, while the morphology and the thermal stability were examined by SEM, DMA, and TGA. The heating’s profile was studied as functions of iron content and induction time. It was found that the time required to complete curing was reduced and the curing temperature was controlled by varying the iron content and induction time. In addition, the thermal stability and the swelling behavior of the prepared composites were enhanced in comparison with the conventional PDMS and thus offer a promising route to obtain thermally stable composites.

## 1. Introduction

Poly(dimethylsiloxane) (PDMS) composites have a wide range of applications as elastomers, adhesives, microfluidic chips, lubricating fluids, and in bio applications [1,2,3]. PDMS based systems are fabricated by molding, casting or soft lithography [4]. PDMS have been shown to be very useful to generate shear fields to align block copolymer patterns [5,6,7]. PDMS is a highly viscous flowing liquid, which also possesses optical transparency, non-flammability, non-toxicity, and biocompatibility for biological assays [8]. PDMS is a heat promoted curable polymer which always comes in a two-part kit consisting of a prepolymer component and a cross-linker component. The curing agent is added to PDMS to obtain elastomeric materials. In the case of PDMS elastomers, one of the main methods of their vulcanization is the hydrosilation reaction, which proceeds between vinyl terminated PDMS chain ends with silicon-hydride cross-linker. There are several catalysts as palladium, peroxides, and azodinitriles are used to activate the curing reaction. In addition, acceleration of the hydrosilation reaction occurs by heating, ultraviolet (UV), or by gamma ray irradiation [9]. Curing by UV has been prepared to avoid exposure to high curing temperatures, but overall the penetration of UV cured system is low compared to heat cured systems, which remain the preferred system [10,11]. Heating is normally used in the fabrication process, including curing and bonding. Currently, the curing techniques of PDMS elastomers by the hydrosilation reaction are very well developed. The curing condition for the pristine PDMS is approximately two days at room temperature, 45 min at 100 °C, 20 min at 125 °C, or 10 min at 150 °C [12]. It has been reported that, cross-linked PDMS elastomers were fabricated at different temperatures [13,14,15]. The PDMS matrix does not need stabilizer because of its low glass temperature and intrinsic stability. Although unmodified PDMS has good flexibility, it still has a low initial temperature for thermal degradation and cannot meet the service requirement in the industry as heat resistance elastomers. To overcome this defect, iron reinforcing filler was introduced into the PDMS matrix by physical blending. Modification by iron as magnetic filler into PDMS matrix has been used in different application as magneto rheological fluids and electromagnetic wave absorbers [16,17,18]. The interest in magnetic induction heating in solving many of the problems facing curing has been rapidly growing, boosted by the search for minimizing the energy required, time consumed, and cost, so that the curing process can be completed with minimal energy in shorter time for large production runs [19]. Materials based on silicon oxide show high thermal protective behavior [20,21]. Magnetic iron particles transform the energy of the magnetic field into heat, which is consequently used as thermal source for the curing process as a simple and more cost-effective approach and for the cure of polyurethane rubbers, plastics, and silicone rubbers [22]. Magnetite-PDMS composites were prepared within 6 min by magnetic induction heating process, where magnetite nanoparticles were used as self-heating particles [23]. Controlling and following the reactions involved in the curing process of iron-PDMS composites has not been previously studied using induction heating. Additionally, inexpensive micron-scale iron powders are commercially available and do not require any additional preparation. Hence, it is important to study the effect of induction heating on the curing rate for composites to fill gaps in our knowledge. The aim of this work is to investigate the impact of iron as self-heating particles in the curing control of Fe-PDMS composites by induction heating. The effect of gas atmosphere type and iron content on the thermal degradation of PDMS were studied and the morphology of the prepared composites was characterized by dynamic mechanical analysis (DMA), thermogravimetric analysis (TGA), and scanning electron microscopy (SEM), respectively. In addition, the specific absorption rate (SAR) and heating rate were measured as a function of iron content and time of induction.

## 2. Material and Methods

### 2.1. Materials

PDMS Sylgard 184 is supplied as a two-part kit consisting of pre-polymer (silicone elastomer base) and cross-linker (184 silicone elastomer curing agent) components (Dow Corning Corporation, Midland, MI, USA) and iron powder (size 5–9 μm) with CAS no. 7439-89-6 was purchased from Sigma-Aldrich (St. Louis, MO, USA).

### 2.2. Preparation of Cured PDMS at Room Temperature

Pre-polymer (silicone elastomer base) and cross-linker (184 silicone elastomer curing agent) were stirred uniformly with a 10:1 weight ratio. The air bubbles were removed by placing the mixture in vacuum and curing at room temperature for 48 h.

### 2.3. Preparation of Fe-PDMS Composites via Induction Heating

The same mixture procedure of PDMS is followed. Then 5, 10, and 15 wt % of iron powder was added to PDMS to form homogenous mixtures of 5%, 10%, and 15% Fe-PDMS respectively. These mixtures were then stirred uniformly and bubble removal by vacuum was carried out. The curing process takes place under a certain time of induction with constant frequency (142 kHz) and power (1 kW). The temperature of a composition under curing was measured by infrared thermometer.

### 2.4. Swelling in PDMS Samples 

The weight of the prepared PDMS was recorded before immersion into a solvent of triethylamine. Then, the weight gain of the PDMS samples was recorded after 1 h, after removal from the solvent and rapid drying, they were weighed on a mass balance.

Degree of swelling (S) = wt_1_ − wt_2_/wt_1_, where wt_1_ and wt_2_ are the weights of dry and swollen PDMS, respectively.

### 2.5. Characterizations

Fourier transform infrared (FTIR) spectroscopy was performed by a Tensor 27 Infrared spectrometer (Bruker, USA). Thermogravimetric analysis (TGA) was measured by TA Instruments Q500at a heating rate of 10 °C/min. Microscopy images were obtained through scanning electron microscopy (SEM) using a Zeiss ULTRA Plus field-emission SEM equipped with a Schottky cathode. The images were analyzed using Smart SEM software v5.05 (Zeiss, Oberkochen, Germany) for imaging operated at 1.5 kV. Strain temperature was measured by Q800 dynamic mechanical analyzer (DMA) (TA Instruments, Elstree, UK). The dimension of the films used for the measurements of mechanical and swelling behaviors was 2 ± 0.1 mm wide and 0.2 ± 0.08 mm thickness.

## 3. Results and Discussion

### 3.1. Heating Properties

**Figure 1:**
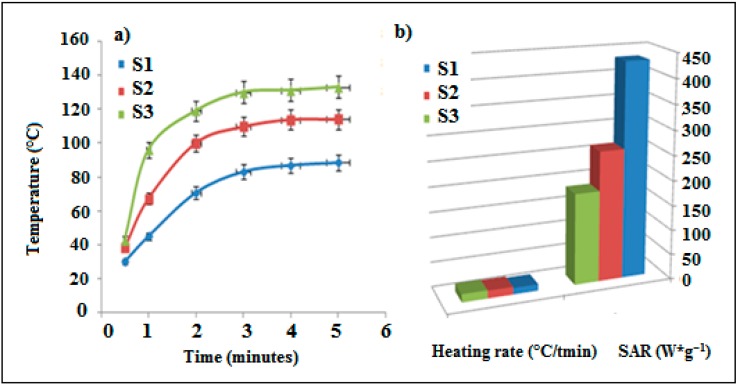
(**a**) Heating properties and (**b**) SAR (W·g^−1^) and heating rate (°C/min) of samples, S1 (PDMS composite with 5 wt % of Fe), S2 (PDMS composite with 10 wt % of Fe) and S3 (PDMS composite with 15 wt % of Fe).

Under of an alternating magnetic field, iron is observed to heat as a result of losses occurring as a result of the internal rotation of the magnetization and the rotation of the particles in a viscous medium. Uniform is necessary to ensure an even heat distribution within the polymer matrix during the curing procedure [24]. The heat generated from samples was evaluated by exposing iron in PDMS to an alternating current (AC) magnetic field for certain time. The reaction of hydrosilation was started by the addition of a Si-H bond across a double bond and the crosslinked silicone polymers become formed. The comparative iron concentrations (5, 10, and 15 wt %) against the exposure time in an AC field with a frequency (142 kHz) and power (1 kW) were investigated (Figure 1). In induction heating curves of samples, it is seen that the heating temperature rises with increasing time until reaches equilibrium temperature, saturated temperature, which is basically after 3 min. It is observed that the maximum temperature induced in the composites increased with the concentration of iron particles. At a saturated temperature, the heating rate (temperature (°C)/time (min)) becomes equal to the cooling rate. The saturated temperatures measured for mixtures with iron contents of 5, 10, and 15 wt % were 85, 115, and 130 °C, respectively. In Figure 1, as the amount of iron increases, the heating rate also increases and the samples are getting heated faster by magnetic field. The rates of heat were 12.91, 15.65, and 16.45 °C/min for iron content 5, 10, and 15 wt %, respectively.

Specific absorption rate (SAR) is the energy amount converted into heat per time and mass (watt per gram) [25]. SAR is calculated using the following Equation (1).

SAR = (C/m) × (dT/dt)
(1)

where dT/dt is the initial slope of the time-dependent temperature curve, Linear relations in 0–3 min intervals are assumed. C is the volumetric specific heat capacity of the sample solution (4.186 J/(g·°C) for water) and m is the mass fraction of Fe particles in composites (wt). The SAR of iron particles in an external AC magnetic field can be attributed to two kinds of power loss mechanisms; Néel relaxation and Brownian relaxation. It was observed that the highest SAR was 432.33 W·g^−1^ and obtained with lower iron content 5 wt %. Additionally, the lowest SAR was 120 W·g^−1^ for iron content 15 wt % (Figure 1). This can be attributed to increasing the amount of iron would decrease the distance between adjacent particles. Néel relaxation is affected by the interaction of inter-particle and the behavior of the inter-particle dipole–dipole interactions. Brownian relaxation is not much sensitive to the concentration of iron moments because the hydrodynamics in the nature of inter-particle force, which leads to reducing the SAR value [26]. In another report the highest SAR value was 36.78 W·g^−1^ for Fe_3_O_4_/PDMS composite. The contribution of heating capacity was 66.67% of Brownian loss obtained with magnetite content 20 wt % [23].

### 3.2. Curing Process of PDMS Composites 

Conventional PDMS is prepared by mixing a 10:1 weight ratio of pre-polymer and cross-linker at room temperature, the liquid mixture of these two parts will become a solid, cross-linked elastomer in two days. The curing and crosslinking of PDMS system is based on the reaction between silicon-hydride groups (Si–H) in the cross-linker and terminal vinyl groups (Si–CH=CH_2_) in the monomer to form an end-linked network with no leaving groups or by-products [27]. 

**Figure 2:**
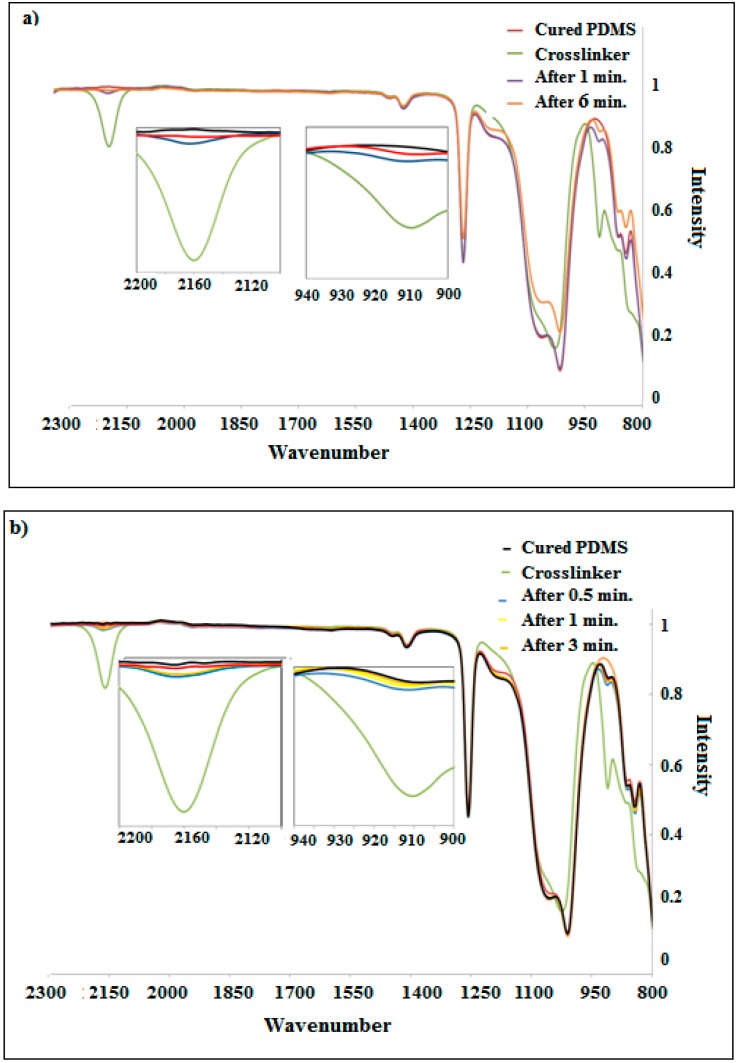

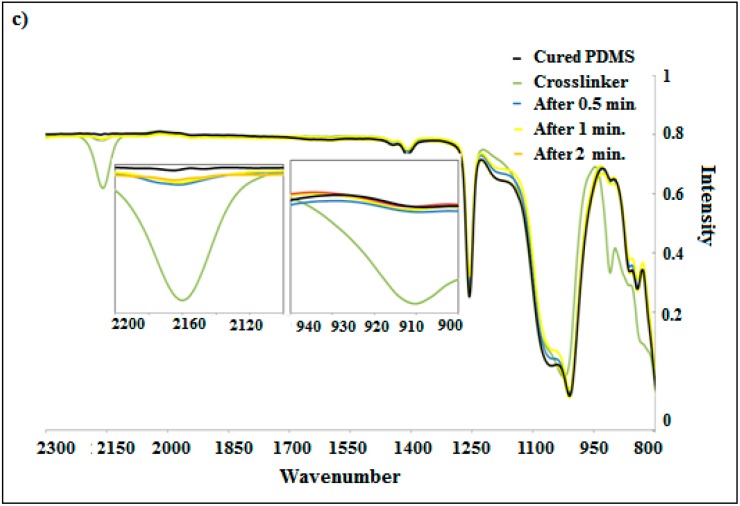
IR of consuming crosslinker at peaks 910 and 2160 cm^−1^ with iron content in the composites (**a**) 5 wt %; (**b**) 10 wt %; and (**c**) 15 wt %.

The effect of the induction heating process on the cross-linking and curing of PDMS systems was investigated by FTIR. IR fingerprint regions for functional groups of interest in hydrosilation curing silicone matrix are followed. It shows the conversion curves of the consumed (–Si–H) groups of the cross-linker as a function of time. Therefore, it is of great interest to follow and control the reactions involved in the curing processes. The impact of iron content and induction time via magnetic induction heating were studied. In Figure 2, the main two peaks are observed at 910 and 2160 cm^−1^, denoting the presence of the crosslinker (–Si–H) that is used to monitor and control the cross-linking reaction and the curing process. Additionally, the low toughness of PDMS lets the microsphere rupture and release the cross-linker into the surrounding medium, thereby initiating the resin cure reaction when enough magnetostrictive force is applied. The cross-linking reaction shows the gradual decrease of the total integrated area of the (–Si–H) by time. This reveals that (–Si–H) groups are getting consumed during the formation of PDMS composites. In Figure 2, it can be seen that the peak for curing PDMS is nearly linear, which means the complete consumption of cross-linker is obtained. In addition, a higher rate of conversion paired to a high percentage of iron is observed. When the reaction is performed via induction heating with the lowest amount of iron for 6 min using 5 wt % of Fe, the conversion levels of almost 94.1% virfication effect hindered the mobility of the polymeric chains. A large number of untreated vinyl groups are trapped in the polymer network. In contrast, by increasing the iron content to 15 wt %, the reaction becomes faster and a higher vinyl group conversion is reached 2 min later, a vinyl group conversion of 92.6% is obtained. It becomes evident that the presence of iron as a filler leads to a progressive increase of the hydrosilation reactivity. The total cure time depends on the vulcanization temperature. As the iron content in the composite increases, the heating rate of the samples increases and, as a consequence, the rates of PDMS vulcanization increase. In this study, it is found that the rate of curing can be modified and controlled by a variety of iron content to achieve a desired working time. In addition, the time required for complete curing for Fe-PDMS is reduced compared with the conventional PDMS.

**Figure 3:**
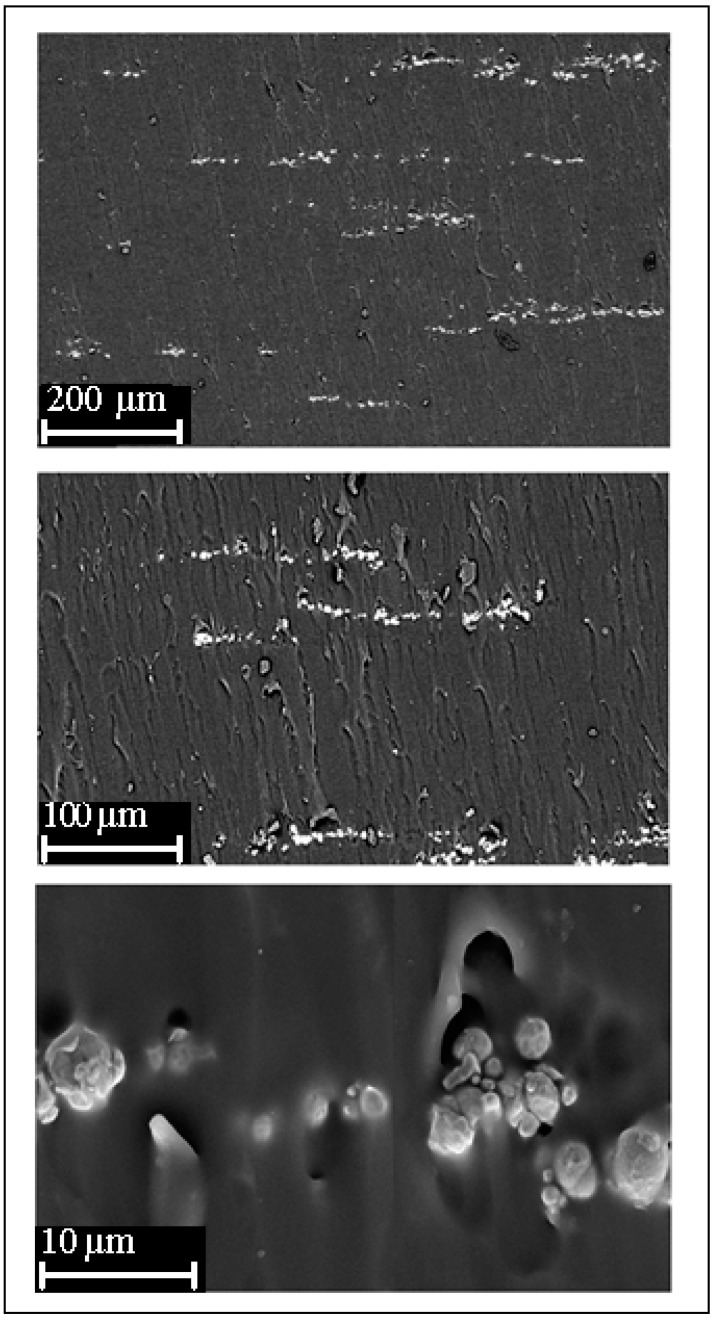
SEM of Fe-PDMS composites.

**Figure 4:**
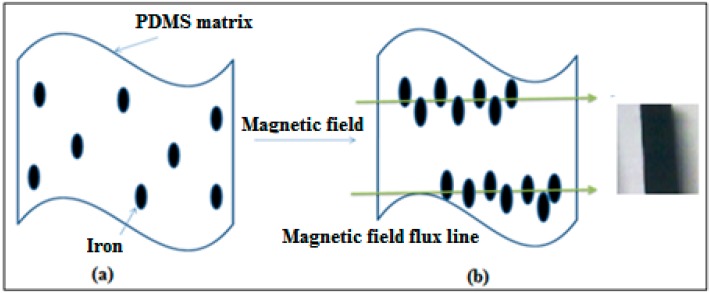
Illustrated schematics and photo of Fe-PDMS composite.

For the distribution of Fe in polymer matrices to have a great effect on thermal properties of resultant composite systems, it is necessary to know the dispersions of Fe in our composites. The morphology of the prepared 10% Fe-PDMS composites, which were prepared by magnetic induction heating, are analyzed by scanning electron microscopy (SEM). The distribution of iron particles as filler and light particles were studied in the PDMS matrix (Figure 3). Heating by application of a magnetic field lead to appreciable variation in the structure of composites. The images show that the iron particles disperse randomly in the PDMS matrix. It is remarkable that, by applying a magnetic field, the particles of iron tend to move to minimize the magnetostatic energy into a stable configuration. In addition, iron particles are aligned parallel quickly in the direction of the magnetic field. Sphere columns are magnetized in the same direction and the magnetic pole interactions between the spheres chain cause them to repel each other until an equilibrium spacing is reached. Finally, it forms long chain-structured particles, which then aggregate to form columns as illustrated in Figure 4. This behavior of formation columns was previously reported by Calabro et al. [28]. In another report, the PDMS shell allows the magnetically responsive particles to respond to an applied magnetic field by distorting the shape of the microsphere such that the shell stretches in the direction of the applied magnetic field [29]. The aggregation and an orientation of Fe particles affect the mechanical property and the swelling behavior of the obtained PDMS elastomer, in good agreement with that of the literature [30].

### 3.3. Swelling Behavior towards Triethylamine

Investigation of the swelling of PDMS with solvents is crucial in designing PDMS based microfluidic devices. A solvent with solubility parameter close to that of PDMS is a good solvent and can cause a higher degree of swelling. For example, triethylamine has a solubility parameter of 7.5, while the solubility parameter for PDMS is 7.3 cal^1/2^ cm^−3/2^. PDMS is commonly known as a polymer that swells with non-polar solvents due to empty networks in the polymer. The behavior of PDMS towards triethylamine is highly interesting. The swelling (%) behavior of the prepared composites towards triethylamine was investigated. The swelling percentages were found to be 34.7%, 43.2%, 63%, and 75% for pure, 5, 10, and 15 wt %, respectively. Fe content affect the degree of crosslinking of the obtained PDMS films and consequently, the swelling behavior might be also affected by the degree of crosslinking of PDMS films.

### 3.4. Effect of Temperature on the Strain Rate

**Figure 5:**
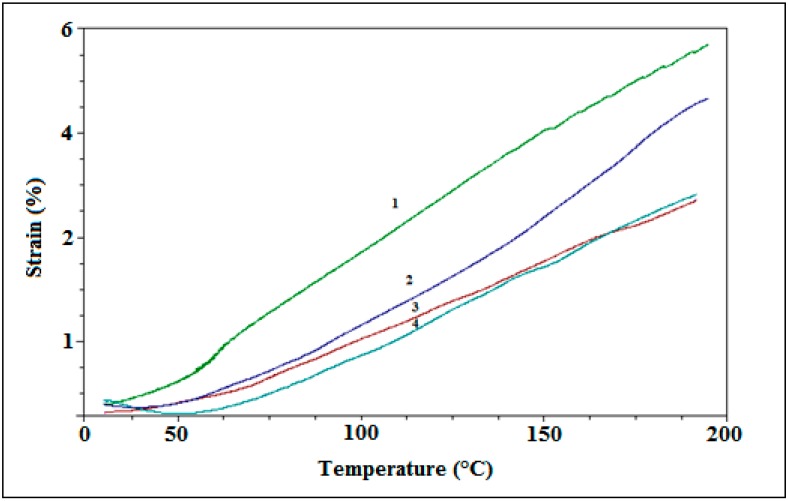
Strain-temperature of (**1**) pure PDMS; (**2**) 5% Fe-PDMS composite; (**3**) 10% Fe-PDMS composite; and (**4**) 15% Fe-PDMS composite.

The effect of temperature on the strain rate was studied by dynamic mechanical analyzer (DMA). Strain-temperature curves of pure PDMS and Fe-PDMS composites were shown in Figure 5. Pure PDMS shows highest deformation by increasing of temperature. In contrast, with Fe-PDMS composites, the large interaction between PDMS and iron particles restricted the heat deformation of PDMS. It was obvious that, as the amount of iron increases, the strain decreases and the samples see a reduction in deformation by temperature.

### 3.5. Thermal Stability of PDMS Composites 

#### 3.5.1. Effect of Iron Content on Thermal Stability of PDMS Composites

**Figure 6:**
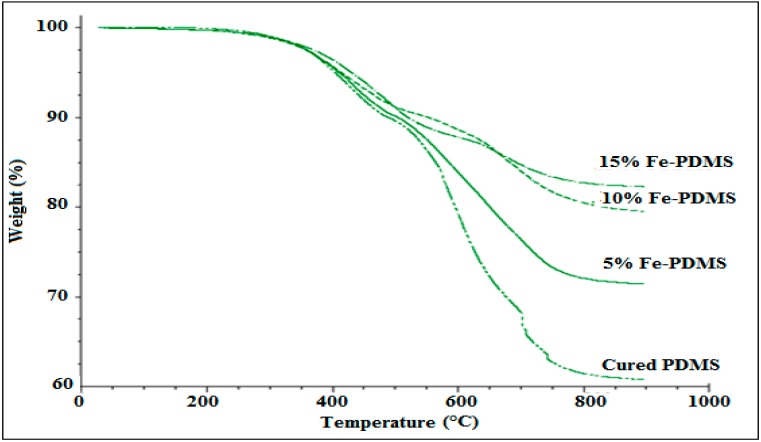
TGA of cured PDMS and PDMS composites as a function of iron content under nitrogen.

The evaluation and the impact of iron content on the thermal stability of Fe-PDMS composites in comparison with the pure PDMS were investigated by thermogravimetric analysis (TGA). Typical TGA weight loss of pure PDMS and Fe-PDMS at different ranges of temperatures when heated from 25 to 900 °C under nitrogen (Figure 6). Weight loose until 400 °C is mainly related to loss of water and physical adsorbed layer. Filling of Fe could hinder the movement of volatiles to enhance the thermal stability of PDMS composites. Above 400 °C, it can be observed that the temperatures of initial decomposition for Fe-PDMS composites shifts to higher values compared with pure cured PDMS, which indicates the presence of iron as a filler enhances the thermal stability of the PDMS matrix in Fe-PDMS composites. In addition, the quantity of the residue increases with the increase of iron content in the composites. The residue values were 61%, 72.5%, 81%, and 83.5% for pure, 5, 10, and 15 wt % composites, respectively. The higher quantity of the residue of Fe-PDMS composites could be attributed to the presence of inorganic compound of iron in the samples which sustain even at high temperatures. 

#### 3.5.2. Effect of Gas Atmosphere Type on Thermal Stability of PDMS Composites

**Figure 7:**
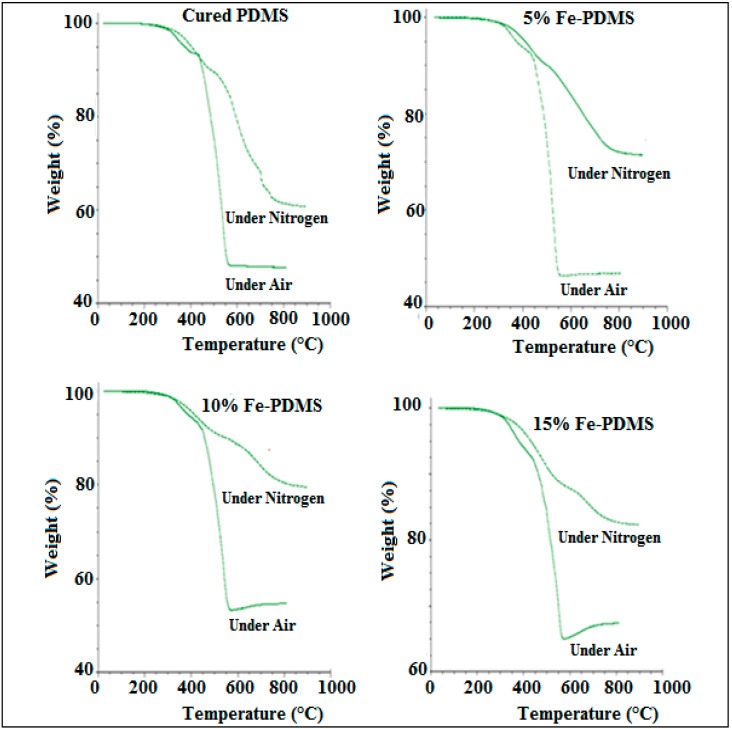
TGA of cured PDMS and PDMS composites as a function of gas atmosphere.

The thermal behavior of the prepared composites in the presence of a different gas atmosphere, namely, nitrogen, and air were studied (Figure 7). In the presence of air, the degradation of PDMS starts at lower temperatures. It was observed a sharp weight loss at 450–600 °C due to the presence of the catalytic effect of oxygen, which increase the de-polymerization of PDMS [31]. In the case of nitrogen, the samples showed continuous slight weight loss in stages till reaching a temperature of 750 °C. It was reported that thermal degradation occurs through the de-polymerization of PDMS with the solid residue of SiO_2_ [32,33]. In another report, it was suggested that the de-polymerization of PDMS through the breaking of Si-O bonds in the PDMS polymer chain [34]. Above 750 °C, the samples become stable and the quantity of the residue increases with the increase of iron content in the composites. Evaluation of composites stabilities of with respect to the iron content and to the type atmosphere has confirmed the thermal stabilizing impact of iron and the catalytic activity of oxygen, respectively. The enhanced thermal stability of the composites compared with the conventional PDMS shows promise as a thermally stable elastomer. High stability PDMS is suitable for processing various biochemical reaction chips. For this purpose, it is important to obtain homogeneous with fully cross-linked PDMS, which will have good stability when subjected to high temperatures.

## 4. Conclusions

In this work, we have investigated the curing behavior and the heating properties in the preparation of Fe-PDMS by magnetic induction heating. We have established that the curing rate can be controlled by a variety of iron contents and induction times. The thermal stability of the composites has revealed that the increase of the decomposition onset temperatures is associated with PDMS degradation strongly depend on the iron particle. The thermal stability, strain, and swelling behavior of the prepared composites are enhanced in comparison with the conventional PDMS. Thus offering a promising route for their use as thermal composites in applications where higher heat resistance is required.

## References

[1] Allan D., Radzinski S.C., Tapsak M.A., Liggat J.J. (2016). The thermal degradation behaviour of a series of siloxane copolymers—A study by thermal volatilisation analysis. Silicon.

[2] Friend J., Yeo J. (2010). Fabrication of microfluidic devices using polydimethylsiloxane. Biomicrofluidics.

[3] Carugo D., Lee J.Y., Pora A., Browning R.J., Capretto L., Nastruzzi C., Stride E. (2016). Facile and cost-effective production of microscale PDMS architectures using a combined micromilling-replica moulding (μMi-REM) technique. Biomed. Microdevices.

[4] Soleymania M., Edrissi M. (2016). Synthesis of bilayer surfactant-coated magnetic nanoparticles for application in magnetic fluid hyperthermia. J. Disp. Sci. Technol..

[5] Qiang Z., Zhang L., Stein G.E., Cavicchi K.A., Vogt B.D. (2014). Unidirectional alignment of block copolymer films induced by expansion of a permeable elastomer during solvent vapor annealing. Macromolecules.

[6] Qiang Z., Zhang Y., Wang Y., Bhaway S.M., Cavicchi K.A., Vogt B.D. (2015). Highly aligned, large pore ordered mesoporous carbon films by solvent vapor annealing with soft shear. Carbon.

[7] Qiang Z., Ye C., Lin K., Becker M.L., Cavicchi K.A., Vogt B.D. (2016). Evolution in surface morphology during rapid microwave annealing of PS-b-PMMA thin films. J. Polym. Sci. Part B.

[8] El-Molla S., Albrecht A., Cagatay E., Mittendorfer P., Cheng G., Lugli P., Salmerón J.F., Rivadeneyra A. (2016). Integration of a thin film PDMS-based capacitive sensor for tactile sensing in an electronic skin. J. Sens..

[9] Brook M.A. (2000). Silicon in Organic, Organometallic and Polymer Chemistry.

[10] Marchi S., Sangermano M., Meier P., Kornmann X. (2016). UV-cured silicone composites obtained via hydrosilation and in-situ generation of inorganic particles. Polym. Eng. Sci..

[11] Marchi S., Sangermano M., Meier P., Kornmann X. (2014). Preparation and characterization of PDMS composites by UV-hydrosilation for outdoor polymeric insulators. Polym. Compos..

[12] (2005). Dow Corning Product Information, “Information about Dow Corning^®^ brand Silicone Encapsulants”. http://businessdocbox.com/Green_Solutions/67460800-Information-about-dow-corning-brand-silicone-encapsulants.html.

[13] Quan S.X. (1989). Properties of post-cured siloxane networks. Polym. Eng. Sci..

[14] Prabowo F., Wing-Keung A.L., Shen H.H. (2015). Effect of curing temperature and cross-linker to pre-polymer ratio on the viscoelastic properties of a PDMS elastomer. Adv. Mater. Res..

[15] Lei K.F., Lee K.F., Lee M.Y. (2012). Development of a flexible PDMS capacitive pressure sensor for plantar pressure measurement. Microelectron. Eng..

[16] Lim S.T., Cho M.S., Jang I.B., Choi H.J. (2004). Magnetorheological characterization of carbonyl iron based suspension stabilized by fumed silica. J. Magn. Magn. Mater..

[17] Zhang B., Feng Y., Xiong J., Yang Y., Lu H.X. (2007). Microwave absorbing properties of deaggregated flake-shaped carbonyl-iron particle composites at 2–18 GHz. IEEE Trans. Magn..

[18] George P.A., Hui W., Rana F., Hawkins B.G., Smith A.E., Kirby B.J. (2008). Microfluidic devices for terahertz spectroscopy of biomolecules. Opt. Express.

[19] Abdou M.I., Abuseda H. (2016). Upgrading offshore pipelines concrete coated by silica fume additive against aggressive mechanical laying and environmental impact. Egypt. J. Petrol..

[20] Fujimoto K., Shioya T., Satoh K. (2002). Mechanical properties of silicon impregnated C/C composite material at elevated temperature. Adv. Compos. Mater..

[21] Arabli V., Aghili A. (2015). The effect of silica nanoparticles, thermal stability, and modeling of the curing kinetics of epoxy/silica nanocomposites. Adv. Compos. Mater..

[22] Abd El-Wahab H., Saleh T.S., Zayed E.M., El-Sayed A.S., Assaker R.S.A. (2015). Synthesis and evaluation of new anti-microbial additive based on pyrimidine derivative incorporated physically into polyurethane varnish for surface coating and into printing ink paste. Egypt. J. Petrol..

[23] Darwish M.S., Stibor I. (2015). Preparation and characterization of magnetite PDMS composites by magnetic induction heating. Mater. Chem. Phys..

[24] EL-Sukkary M.M., Ismail D.A., El Rayes S.M., Saad M.A. (2014). Synthesis and evaluation of some derivatives of polysiloxanes. Egypt. J. Petrol..

[25] Darwish M.S., El-Sabbagh A., Stibor I. (2015). Hyperthermia properties of magnetic polyethylenimine core/shell nanoparticles: Influence of carrier and magnetic field strength. J. Polym. Res..

[26] Zhang J., Boyed C., Luo W. (1996). Two mechanisms and a scaling relation for dynamics in ferrofluids. Phys. Rev. Lett..

[27] Liu M., Sun J., Sun Y., Bock C., Chen Q. (2009). Thickness-dependent mechanical properties of polydimethylsiloxane membranes. J. Micromech. Microeng..

[28] Calabro J.D., Huang X., Lewis B.G., Ramirez A.G. (2010). Magnetically driven three-dimensional manipulation and inductive heating of magnetic-dispersion containing metal alloys. Proc. Natl. Acad. Sci. USA.

[29] Carlson J.D. (2012). Magnetically Curable Composition and Magnetic Cure Process. U.S. Patent.

[30] Hajsz T., Csetneki I., Filipcsei G., Zrinyi M. (2006). Swelling kinetics of anisotropic filler loaded PDMS networks. Phys. Chem. Chem. Phys..

[31] Esteves A.C., Brokken-Zijp J., Laven J., de With G. (2010). Light converter coatings from cross-linked PDMS/particles composite materials. Prog. Org. Coat..

[32] Camino G., Lomakin S.M., Lageard M. (2002). Thermal polydimethylsiloxane degradation. Part 2. The degradation mechanisms. Polymer.

[33] Fujimoto S., Ohtani H., Tsuge S. (1988). Characterization of polysiloxanes by high-resolution pyrolysis-gas chromatography-mass spectrometry. Fresenius J. Anal. Chem..

[34] Camino G., Lomakin S.M., Lazzari M. (2001). Polydimethylsiloxane thermal degradation Part 1. Kinetic aspects. Polymer.

